# The TNF-α System: Functional Aspects in Depression, Narcolepsy and Psychopharmacology

**DOI:** 10.2174/157015908785777238

**Published:** 2008-09

**Authors:** Mark Berthold-Losleben, Hubertus Himmerich

**Affiliations:** Department of Psychiatry and Psychotherapy, RWTH Aachen University, Pauwelsstraße 30, 52074 Aachen, Germany

**Keywords:** TNF-α, TNF receptor, depression, antidepressant, antipsychotic.

## Abstract

Changes of the tumor necrosis factor-alpha (TNF-α) system have been shown to be involved in the development of psychiatric disorders and are additionally associated with changes in body weight as well as endocrine and metabolic changes in psychiatric patients.

TNF-α might, for example, contribute to the pathogenesis of depression by an activation of the hypothalamo-pituitary-adrenocortical (HPA) axis, an activation of neuronal serotonin transporters and the stimulation of the indoleamine 2,3-dioxygenase which leads to tryptophan depletion. On the other hand, during an acute depressive episode, an elevated HPA axis activity may suppress TNF-α system activity, while after remission, when HPA axis activity has normalized the suppression of the TNF-α system has been shown not to be apparent any more.

In narcoleptic patients, soluble TNF receptor (sTNF-R) p75 plasma levels have been shown to be elevated, suggesting a functional role of the TNF-α system in the development of this disorder.

Additionally, psychotropic drugs influence the TNF-α system as well as the secretion and the effect of hormones which counteract or interact with the TNF-α system such as the intestinal hormone ghrelin. However, only preliminary studies with restricted sample sizes exist on these issues, and many open questions remain.

## INTRODUCTION

The brain and the immune system are the two major adaptive systems of the body. Both influence and regulate each other. Factors stemming from the immune system, such as cytokines and chemokines, and factors derived from the central nervous system, for example hormones, neurotransmitters and neuropeptides, have an influence on both, the brain and the immune system. And there is growing evidence that infectious and immune factors may contribute to the pathogenesis of neurodevelopmental disorders including autism, mental retardation, and schizophrenia [38]. More specifically, more and more support for the hypothesis is found that the tumor necrosis factor (TNF)-α producing macrophages play an important role in neurodevelopment and in the pathophysiology of various neuropsychiatric conditions [19].

The central nervous system affects the immune system mainly through the neuroendocrine outflow *via* the pituitary, and through direct neuronal influences *via* the sympathetic, parasympathetic, peptidergic and sensory innervation of peripheral tissues. Alternatively, certain cytokines such as interleukin (IL)-1, IL-6 and TNF-α released during an immune response and other processes the immune system is involved in modulate brain activity. These cytokines act on the brain *via* a fast transmission pathway involving primary afferent nerves innervating the bodily site of inflammation and a slow transmission pathway involving cytokines originating from the choroid plexus and circumventricular organs and diffusing into the brain parenchyma by volume transmission. They alter neurotransmitter networks activity and, thus induce sleepiness, fatigue, loss of appetite and decreased libido. These symptoms have been described as “sickness behaviour” and are related to the behavioral changes of depression [15].

Among the different ways of bidirectional influence between the immune system and the central nervous system, one cytokine has recently attracted a lot of scientific interest: TNF-α. This review focuses on the TNF-α system, although other cytokines do also contribute to the interaction between the brain and the immune system and, in part, amplify the effects of TNF-α.

## THE TNF-α SYSTEM

TNF-α is a 185 amino acid glycoprotein hormone, which was isolated in 1975 by Carswell *et al*. as a soluble factor released by host cells that caused necrosis of a transplanted tumor [9]. Once TNF-α was cloned in 1985, the involvement of this cytokine in infectious disease pathology was pursued by a number of groups. By the late 1980s excess TNF-α production was proposed to be central to acute systemic viral diseases. This family of cytokines is now at the centre of investigations to understand the mechanisms of acute systemic viral diseases, including influenza and the hemorrhagic viral diseases. With its implication as the master regulator of other inflammatory cytokines in the synovial membrane, TNF-α has also become the major cytokine in the pathogenesis of chronic inflammatory disease. Its neutralization has proven to be a potent treatment for rheumatoid arthritis and Crohn's disease [12].

TNF-α is a multifunctional signaling molecule with important functions in inflammation and apoptosis [21]. It plays a significant role in the defense against viral, bacterial and parasitic infections as well as in autoimmune disorders and energy homeostasis [18]. TNF-α is released by monocytes / macrophages and other white blood cells, the endothelium, the adipose tissue and several other tissues. It is considered to be one of the most important factors of inflammation and cachexia [67]. In conclusion, it has a wide range of functions within the body, including a role in immune response, inflammation, apoptosis, and energy homeostasis. It is involved in several regulatory processes within the body [26].

The action of TNF-α at the cellular level is mediated by two cell surface receptors, TNF-R p55 and TNF-R p75. TNF-R p55 is expressed in most tissues, whereas expression of TNF-R p75 is highly regulated and typically found in cells of the immune system. Accordingly, in the vast majority of cells, TNF-R p55 appears to be the key mediator of TNF-α signaling, whereas in the lymphoid system, TNF-R p75 seems to play a major role [101]. When bound to TNF-α, the TNF-Rs transduce growth regulatory signals into the cell. *Via* these receptors, TNF-α is mitogenic in normal cells; however, TNF-α initiates apoptosis in transformed cells causing DNA fragmentation and cytolysis. Whether TNF-α induces cell differentiation or apoptosis *via* the TNF-Rs depends on the signaling pathway activated within the cell. The TNF-α-induced survival pathway seems to be mediated by the transcription factor nuclear factor-kappaB (NF-KB), as recent studies have demonstrated that cells in which the NF-KB signaling pathway is blocked are more likely to undergo apoptosis in response to TNF-α [72].

In cases in which TNF-α induces apoptosis, it has been shown that one of the crucial intracellular signaling event is the sequential activation of caspases, a family of cysteine proteases [1].

The membrane-bound TNF-Rs can be proteolytically cleaved from the cell membrane by the proteolytic action of a disintegrin metalloproteinase called TNF-α converting enzyme (TACE) [77]. Therefore, soluble TNF-Rs (sTNF-Rs) are soluble variants of the extracellular domains of their membrane-bound form. Although soluble forms of cytokine receptors such as sTNF-R p75 are thought to control cytokine activity *in vivo* by inhibiting the ability of cytokines to bind to their membrane receptors and thus inhibiting a biological response, elevated plasma levels of sTNF-R p75 indicate an inflammatory process in several diseases, for example rheumatoid arthritis [20]. Fig. (**1**) shows a simplified scheme of the TNF-α system and the components described above.

## METABOLIC AND NEUROENDOCRINE ASPECTS OF THE TNF-α SYSTEM

Obesity is associated with a chronic low-grade inflammation and an increased abundance of macrophages in the white adipose tissue. The macrophages seem to lead to a chronic activation of the innate immune system. This low-grade inflammation results can subsequently lead to insulin resistance, impaired glucose tolerance and even diabetes [5].

The energy balance and weight regulation, the control of appetite and feeding are based on a complex network of hormones, cytokines, neurotransmitters and neuromodulators. One of them is the 28-amino acid peptide hormone ghrelin which regulates feeding behaviour through modulation of expression of the orexigenic peptides, neuropeptide Y and agouti-related protein in the hypothalamus [60, 98]. Ghrelin is secreted in the gastrointestinal tract and correlates inversely with the body mass index and fat percentage [99]. It could be shown that ghrelin administration increases food intake and body weight, while weight loss increases ghrelin levels. Ghrelin is an important factor of energy balance, weight regulation and the control of appetite and feeding, and its dysregulation is assumed to play an important role regarding obesity [14, 47, 60, 98, 108, 109]. Therefore, TNF-α and ghrelin seem to be opponents regarding the hypothalamic regulation of eating behavior as TNF-α has an anorexigenic effect and ghrelin exhibits an orexigenic effect on an hypothalamic level.

Additionally, ghrelin, and its target receptors, the growth hormone secretagogue receptors (GHS-Rs), have been localized to neutrophils, lymphocytes, and macrophages, which suggests that ghrelin may be involved in the process of immune modulation. And indeed, ghrelin has potent anti-inflammatory properties through modulation of secretion of both pro-inflammatory and anti-inflammatory cytokines from macrophages [104]. Overall, ghrelin exhibits anti-inflammatory effects as well as immunoregulatory effects that may be mediated through the GHS-R-1a receptor [116] and through activation of the vagus nerve [110]. It down-regulates pro-inflammatory cytokines and inhibites NF-kappaB activation. E.g. ghrelin could be shown to improve survival rates of rats with sepsis-induced acute lung injury [111], might ameliorate neuropathic pain by diminishing the pro-inflammatory cytokines and regulating pain system [22], and therefore may be a possible anti-inflammatory drug in the future. To conclude, on the level of immune cells, ghrelin also seems to counteract the effect of pro-inflammatory cytokines.

In obesity, the white adipose tissue is characterized by an increased production and secretion of a wide range of inflammatory molecules including TNF-α, which may have local effects on white adipose tissue physiology but also systemic effects on other organs. Interestingly, weight loss is associated with a reduction in the macrophage infiltration of white adipose tissue and an improvement of the inflammatory profile of gene expression. Several factors derived not only from adipocytes but also from infiltrated macrophages probably contribute to the pathogenesis of insulin resistance. Most of them are overproduced during obesity, including leptin, TNF-α, IL-6 and resistin [5]. 

Macrophages exist in separate types of differentiation, but the nature of adipose tissue macrophages is largely unknown. However, in a study of Zeyda *et al*. [114] basal and induced secretion of pro-inflammatory mediators such as TNF-α was even higher in adipose tissue macrophages than in other pro-inflammatory macrophages. 

Therefore, it could be shown by several investigations that body fat mass influences TNF-α plasma levels [64, 66]. However, much less is known about plasma levels of its soluble receptors sTNF-R p55 and sTNF-R p75. In a study with more than 500 randomly chosen adults, plasma levels of TNF-α and also sTNF-Rs correlated significantly with the BMI [31].

As increased pro-inflammatory cytokine levels are known to be associated with cardiovascular disease [25, 41, 81], silent brain infarctions [39], and a worse prognosis after stroke [102], Park *et al*. concluded that the positive associations of obesity and visceral adiposity with elevated cytokine levels suggest the importance of reducing obesity and visceral adiposity to prevent elevations in cytokine levels and associated diseases [65].

Cytokines such as TNF-α link the non-specific immune system to the hypothalamo-pituitary-adrenocortical (HPA) axis: inflammatory cytokines — such as TNF-α and its soluble receptors p55 and p75 — released during infection and inflammation activate the HPA system at the hypothalamic, pituitary, and adrenal level resulting in the release of cortisol as the most important negative feedback signal to prevent an overshoot of the ongoing host defense [42, 89, 96]; glucocorticoids, in turn, suppress the production of pro-inflammatory cytokines. Moreover, a chronically activated HPA axis as occurring during chronic stress results in defective immune system responses to an inflammatory challenge [3, 6, 80, 84]. It could be shown in tumor bearing patients that exogenous TNF-α administration leads to a corticotropin-releasing hormone (CRH), adrenocorticotropic hormone (ACTH) and cortisol release [55], while glucocorticoid administration in healthy subjects suppresses TNF-α production, and the circadian rhythm of endogenous glucocorticoids appears to be inversely correlated with the pro-inflammatory cytokine production [69]. Fig. (**2**) is ment to show the interactions between the TNF-α system and the HPA axis and its negative feedback mechanism.

Additionally, cytokines such as TNF-α exert other effects on the hypothalamic level. The pro-inflammatory cytokines IL-1, IL-6 and TNF-α have been most investigated for their pyrogenic action. The experimental evidence demonstrates the role of these secreted proteins in modulating the fever response. An association between cytokine levels in serum and cerebrospinal fluid and fever, the finding of the presence of cytokine receptors on various cell types in the brain and demonstration of the effects of pharmacological application of cytokines and of their neutralizing antibodies on the fever response and fever studies on cytokine- and cytokine receptor-transgenic models underline the strong influence of cytokines on hypothalamic thermoregulation [13].

Cytokine-induced loss of appetite is consistently observed during cytokine immunotherapy in humans. Investigators have also shown that cytokines induce anorexia when administered peripherally or into the brain. Anorexigenic cytokines by central action include IL-1, IL-6, leptin, IL-8, TNF-α, and interferon (IFN)- γ. Cellular approaches indicate that modulation of hypothalamic activity is involved in cytokine-induced anorexia. Cytokine action involves the modulation of specific neurons that are proposed to participate in the control of feeding, i.e. glucose-sensitive neurons in the lateral hypothalamic area and hypothalamic ventromedial nucleus [71]. Appetite loss and fever lead to lipolysis, lipid mobilization and a reduction of the fatty tissue. 

One condition already mentioned in which TNF-α is produced and secreted into the blood is obesity. Nowadays the concurrence of rich food and physical idleness is an increasing problem and established risk factor for various diseases in affluent countries. Different combinations of obesity, diabetes, hypertriglyceridemia and insulin resistance cause hepatic steatosis, which can trigger necroinflammation and fibrosis. Patients with steatohepatitis exhibit ultrastructural mitochondrial lesions. Mitochondria play a major role in fat oxidation and energy production but also leak reactive oxygen species (ROS) and are the main cellular source of ROS. In patients with steatosis, mitochondrial ROS may oxidize hepatic fat deposits. Lipid peroxidation products impair the flow of electrons along the respiratory chain, which may cause overreduction of respiratory chain components, further increasing mitochondrial ROS formation and lipid peroxidation. Lipid peroxidation, in turn, causes further cytokine induction [68].

## THE TNF- α SYSTEM AND DEPRESSION

Several psychiatric disorders are reported to be associated with alterations of the cytokine system. In this article, hypotheses regarding associations of major depression, an affective disorder, and narcolepsy, a sleep disorder, with the TNF-α system are demonstrated. Additionally, one should keep in mind that the TNF-α system is as well involved in the development of other brain disorders such as multiple sclerosis, Parkinson’s disease and Alzheimer’s disease [26].

Modern neurobiological methods have revealed pathophysiological mechanism associated with depression. The monoamine hypothesis, which was advocated in the 1950s, emphasizes that the deficiency of monoamine neurotransmitters, for example serotonin, brings about depressive symptoms. This theory played an important role in promoting the development of new antidepressants and it is underlined by genetic findings of polymorphisms of serotonin transporter gene associated with depression. Neuroendocrine studies have revealed the HPA axis dysfunctions in depressive patients and increased activity of HPA axis are considered as state marker of depression [93]. Several findings indicate an influence of the cytokine system, of which TNF-α is a part, on serotonin metabolism as well as on the HPA axis.

Alterations in plasma cytokine levels have repeatedly been found in patients suffering from affective disorders [23, 52], and evidence suggests that cytokines may be involved in the development of depression [76]. It has been postulated that the activation of the cytokine system might play a causative role in the depression-related activation of the HPA system [51, 63], and experimental studies applying immune stimulation in humans [78, 79] as well as in rodents [50, 112] showed that immune stimulation induces depression-like signs and symptoms supporting the view that inflammatory cytokines are causally involved in behavioral alterations of patients with depressive disorders.

In contrast, Schuld *et al*. reported data suggesting that chronic HPA system overactivity in depressed patients suppresses the production of inflammatory cytokines [86]. In another study on the mutual influence of the HPA system and the TNF-α system in depressed patients without inflammatory diseases, TNF-α levels were inversely associated with the ACTH response to the combined dex/CRH test and it was concluded that elevated HPA axis activity in acute depression suppresses TNF-α system activity [29]. Therefore, the activated HPA axis in depressed patients seems to suppress the activity of the cytokine system.

As pro-inflammatory cytokines and serotonergic homeostasis have both been implicated in the pathophysiology of major psychiatric disorders, Zhu *et al*. hypothesized that cytokines might also activate neuronal serotonin transporters. This idea would underline the theory of a serotonin deficiency during depression and the pharmacodynamic mechanism of selective serotonin reuptake inhibitors (SSRI) in the treatment of depression, because SSRIs lead to recovery from depression *via* deactivation of serotonin transporters. Indeed, Zhu *et al*. found TNF-α stimulated serotonin uptake in both a rat embryonic raphe cell line and in mouse midbrain and striatal synaptosomes. These results provided evidence that pro-inflammatory cytokines can acutely regulate neuronal serotonin transporter activity. A mitogen-activated protein kinase may be in involved in this mechanism [117].

Pro-inflammatory cytokines such as IL-1 and TNF-α affect the tryptophan metabolism directly or indirectly by stimulating the enzyme indoleamine 2,3-dioxygenase which leads to a peripheral depletion of tryptophan [106]. And the aromatic amino acid tryptophan functions as precursors for the monoamine neurotransmitter serotonin in the brain.

Therefore, we can hypothesize three mechanisms how cytokines may lead to depression or depressive symptoms: the activation of the HPA axis, the activation of neuronal serotonin transporters and the stimulation of the indoleamine 2,3-dioxygenase which leads to tryptophan depletion.

## THE TNF-α SYSTEM AND NARCOLEPSY

Narcolepsy is a disabling sleep disorder characterized by excessive daytime sleepiness, cataplexy, hypnagogic hallucination, and sleep paralysis [62]. Since the discovery of the extremely close association of narcolepsy and the human leukocyte antigen HLA-DR2 [48, 56] it has been suggested that the immune system might play a pathogenic role, because it is known that HLA haplotypes are linked to a number of autoimmune diseases [57]. In human narcoleptics, a dramatic reduction in the number of hypocretin neurons could be observed [16]. Because of the association of narcolepsy with HLA-DR2 it was hypothesized that the loss of these neurons might be caused by an autoimmune process [94].

Furthermore, a small number of studies suggest that certain cytokine-producing genes may predispose to narcolepsy. Hohjoh *et al*. [37] conducted an association study of the TNF-R p75 polymorphisms with human narcolepsy and found that the 196 R allele was significantly more frequent in narcoleptic patients suggesting that this allele is associated with the susceptibility to narcolepsy.

In a sample of 30 narcoleptic patients in comparison to 120 gender- and age-matched and 101 gender-, BMI- and age-matched randomly selected normal controls, sTNF-R p75 levels were consistently elevated in the narcoleptic patients, even if compared to gender-, BMI- and age-matched counterparts. It was concluded that narcoleptic patients show increased plasma levels of sTNF-R p75 suggesting a functional alteration of the TNF-α cytokine system and further corroborating a possible pathogenic role of the immune system in this sleep disorder [28]. One possible hypothesis would be that the activation of the TNF-α system and a sTNF-R p75-mediated activation of cells of the immune system would lead to an autoimmune destruction of hypocretin neurons, which seems to be strongly associated with the development of narcolepsy. This destruction may involve T-helper cells (TH), cytotoxic T cells and lymphocytes.

In conclusion, TNF-α may lead to psychiatric disorders *via* an activation of a neuroendocrine system such as the HPA axis, the activation of neurotransmitter transporters such as the serotonin transporter, the influence on the metabolism of neurotransmitters and the autoimmune destruction of neurons.

## THE TNF-α SYSTEM AND OTHER DISEASES OF THE BRAIN

In addition to its immunological and metabolic properties, TNF-α promotes nerve cell growth and differentiation, and is cytotoxic to oligodendrocytes [54]. The gene that encodes TNF-α is mapped to chromosome 6p21.3–p21.1 [91], a region linked in several studies to Schizophrenia [2, 49, 87, 103].

Thus, TNF-α might be involved in the process of neurodevelopment and neurodegeneration, which could link to the pathogenesis of Schizophrenia by subtle alterations in neuronal number and density [105]. A functional single nucleotide polymorphism within the promoter region of the TNF-α gene, has been related to the pathogenesis of several diseases, including Schizophrenia [107]. TNF-α has pleiotrophic effects on neurons, particularly in glutamate excitotoxicity by inhibiting glial glutamate transporters on astrocytes directly and indirectly [70]. The G-308A polymorphism of the TNF-α gene might be involved in antipsychotic response [112].

The TNF-α system has also been shown to be involved in the development of other brain diseases such as multiple sclerosis (MS). In MS patients, the inflammatory demyelinisation seems to be mediated by the presence of TNF-α leading to myelin and oligodendrocyte destruction [46]. One source of TNF-α production may be the dendritic cells [43]. TNF-α induces neurotoxicity *via* glutamate release from hemichannels of activated microglia in an autocrine manner. This mechanism may contribute to neuronal damage in neurodegenerative diseases including Alzheimer’s disease, Parkinson’s disease, amyotrophic lateral sclerosis and multiple sclerosis [92].

## CHANGES OF THE TNF-α SYSTEM DURING PSYCHOPHARMACOLOGICAL TREATMENT

Weight gain in patients on psychotropic medication is a frequent side effect of prominent clinical relevance, occurring under antipsychotics and antidepressants.

In recent years, studies have been conducted on neuroendocrine and neuroimmunological mechanisms involved in appetite and weight regulation. Regarding these mechanisms, the TNF-α system might play a particular role. All drugs investigated so far that induce weight gain - clozapine, olanzapine, amitriptyline and mirtazapine – also clearly activate the TNF-α system [36, 44, 75, 85]. This activation results in increased plasma levels of soluble sTNF-Rs and TNF-α levels.

Recently, the influence of the mood stabilizer carbamazepine on leptin and the TNF-α system has been investigated, too. Lithium, for example, demonstrated significant immunoregulatory effects by increasing the *in vitro* production of TNF-α in healthy volunteers [52], and the TNF-α and sTNF-R p75 levels have already been shown to be significantly increased cross-sectionally in patients treated with lithium [23].

A longitudinal study on the influence of carbamazepine and lithium on TNF-α plasma levels has been conducted. An increase in the body mass index and in TNF-α and its soluble receptor levels over the 4 weeks of treatment was found in both treatment groups [33].

Is not clear, however, whether the activation of the TNF-α system by psychotropic drugs is the cause or a consequence of weight gain during psychopharmacological treatment. As mentioned above, recent data indicate that white adipose tissue is infiltrated by macrophages, which may be a major source of locally produced pro-inflammatory cytokines [5], in case of increasing body weight.

Activation of the TNF-α system seems to be specific for psychotropic drugs that induce weight gain, since drugs which did not affect or even decreased weight (haloperidol, paroxetine, venlafaxine) did not influence the TNF-α system at all [33, 36, 74, 75, 85]. Moreover, the antidepressant bupropion, which reduces body weight, lowers the production of TNF-α in mice [7]. An overview of the TNF-α and TNF-R production-inducing properties of various psychopharmacological agents is shown in Table **1**.

Abnormal liver function tests are frequently encountered in patients receiving psychopharmacological treatment [17]. All kinds of psychotropic drugs can elevate liver enzyme levels and have the potential to induce liver damage [88]. In a study on liver enzyme levels during psychopharmacological treatment in patients who received a stable psychopharmacological treatment, an increase in aspartate-aminotrans-ferase (ASAT) and alanine-amino transferase (ALAT) plasma levels was found. This increase in ALAT and ASAT levels was most pronounced at week three. Analyzing the changes from baseline to week three, among several other parameters, changes in TNF-α levels were significantly associated with changes in ALAT [32].

This finding suggests that the effects of psychotropic drugs on the TNF-α cytokine system – independent of weight gain – might contribute to early changes in liver enzyme levels. This idea is in line with present evidence already mentioned earlier that cytokines might be involved in liver damage [10, 40, 97]. This might be particularly true for substances such as clozapine that have independent effects on weight regulation and the TNF-α system [4, 35]. But beyond the activation of the TNF-α system, body weight during psychopharmacological therapy seems to be the best predictor for liver enzyme elevation during a treatment period of several weeks [32].

In a small study with 24 depressed patients we sought to determine whether changes in TNF-α and its receptor plasma levels early during antidepressant therapy are predictors of psychopharmacologically induced weight change. Changes of weight during the first week of treatment, but no parameter of the TNF-α system strongly predicted weight change until endpoint of the study. Therefore, changes regarding the TNF-α system do not seem to be a clinically useful predictor of future weight development during psychopharmacological therapy [34], but to know exactly more research needs to be done in this area.

As mentioned above, the effects of the TNF-α system may be modulated or even counteracted by the intestinal hormone ghrelin. Ghrelin recently became a target of research regarding investigations on weight gain during psychopharmacological treatment. 

Regarding antipsychotic treatment for example, it was reported that olanzapine would increase plasma ghrelin levels in patients with schizophrenia [59] and that acute administration of clozapin increases circulating plasma ghrelin levels in rats [58]. Data of other studies, however, did support the hypothesis of a causal involvement of ghrelin in antipsychotic-induced weight gain [30, 82, 95]. However, changes in weight during treatment with antipsychotics may be the cause for changes regarding ghrelin secretion. These interrelations may be elucidated by future research.

Regarding antidepressant treatment, the role of ghrelin seems to be much more complex and the various study results are not sufficient to lead to a common hypothesis for the role of ghrelin in antidepressant treatment. For example, tryptophan – the precursor molecule of serotonin – enhances ghrelin expression and secretion [115], antidepressant therapy with an SSRI may attenuate hypothalamic effects of ghrelin [8], and antidepressant therapy with mirtazapine seems to decrease ghrelin levels [83]. To our knowledge, studies investigating TNF-α as well as ghrelin levels during antidepressant treatment are not available.

## PERSPECTIVES

It is pharmacologically possible to give a TNF-α blocker to a patient. One of these new drugs is etanercept. It can, for example, be used for the treatment against the autoimmune disease psoriasis. Psoriasis has substantial psychological and emotional effects. Therefore, Tyring *et al*. assessed the effect of etanercept on fatigue and symptoms of depression associated with the condition. 618 patients with moderate to severe psoriasis received double-blind treatment with placebo or 50 mg twice-weekly etanercept. The primary efficacy endpoint was a 75% or greater improvement from baseline in psoriasis area and severity index score at week 12. Secondary endpoints included the functional assessment of chronic illness therapy fatigue scale, the Hamilton Depression Rating Scale (HAMD), the Beck depression inventory (BDI), and adverse events. In this study, greater proportions of patients receiving etanercept had at least a 50% improvement in the HAMD or BDI at the endpoint of the study; patients treated with etanercept also had significant and clinically meaningful improvements in fatigue. The improvements in symptoms of depression were less correlated with objective measures of skin clearance or joint pain. The authors concluded that etanercept treatment might relieve fatigue and symptoms of depression associated with this chronic disease [100]. This observation together with the theoretical background reported in this article leads to the hypothesis, that TNF-α blocker might be useful novel antidepressant agents.

One should also consider, whether cytokines could be involved in the development of morphological changes of hippocampus and down regulation of neurotrophin observed in depressive patients. But no investigations are available regarding these associations. 

Recent genetic research points at the importance of caspase genes for the predisposition to major depression [24], and caspases are one intracellular signaling way of TNF-α signaling. Moreover, genetic findings point at the importance of the NF-kappaB-inducing kinase (NIK) in the pathogenesis of bipolar affective disorders. For the role of NIK in TNF-α signaling see Fig. (**1**) [61]. These and further genetic findings may elucidate the way how TNF-α is pathophysiologically involved in the development of affective disorders.

## LIMITATIONS

This review focuses on plasma levels of TNF-α and its soluble receptors. Other inflammatory and anti-inflammatory cytokines act together with TNF-α and should therefore at least be mentioned. We chose the TNF-α system as an issue of this review, because significant findings regarding its involvement in brain disorders - such as depression, narcolepsy, multiple sclerosis, Alzheimer’s and Parkinson’s disease - and psychopharmacology have been reported in several study reports [26]. In the majority of studies regarding the TNF-α system and psychiatric disorders or psychopharmacological treatment, it would have also been useful to determine the concentration of anti-inflammatory cytokines such as IL-10 or IL-4 to assess the degree of imbalance between the inflammatory and anti-inflammatory arms of the immune system, but this issue has not been addressed systematically during psychoimmunological research.

As the majority of the mentioned studies are cross-sectional, caution is recommended regarding conclusions about the causality. The conclusion that elevated cytokine levels may contribute to the pathogenesis of depression is derived from the literature combining the conclusions of several studies, but can not be substantiated from longitudinal studies. Therefore, it would be necessary to strengthen experimental and longitudinal clinical research in animals and humans investigating the effect of cytokines on mood over a certain period of time.

One could argue that the cited studies reported mainly plasma levels of TNF-α and its soluble receptors. This is an important demur. On the other hand, Sorge [90] reported an increase of TNF-α and sTNF-R levels during treatment with clozapine, as it was expected from the observations of increasing plasma levels of TNF-α and sTNF-R levels within the blood. Additionally, Churchill *et al*. [11] reported that systemic application of TNF-α increases TNF-α mRNA levels in the hypothalamus, hippocampus and the somatosensory cortex. Therefore, one can expect that plasma levels of TNF-α correlate with the TNF-α level within the brain.

To conclude, only preliminary studies with restricted sample sizes exist on most of the mentioned issues, and many open questions remain.

## Figures and Tables

**Fig. (1):**
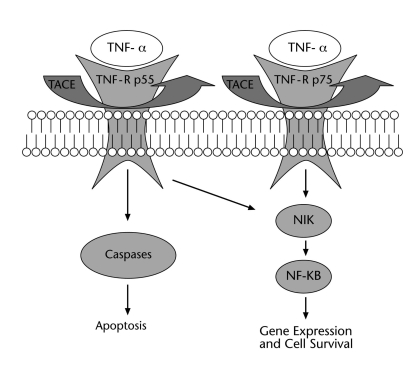
**Simplified scheme of TNF-α signaling.** The action of TNF-α at the cellular level is mediated by the cell surface receptors TNF-R p55 and TNF-R p75. Whether TNF-α induces cell differentiation or apoptosis depends on the signaling pathway activated within the cell. The TNF-α-induced survival pathway seems to be mediated by the transcription factor NF-κB. In cases in which TNF-α induces apoptosis, the crucial intracellular signaling event is the sequential activation of caspases. The membrane-bound TNF-Rs can be proteolytically cleaved from the cell membrane by the proteolytic action of a disintegrin metalloproteinase called TNF-α converting enzyme. Abbreviations: tumor necrosis factor-alpha (TNF-α), TNF receptor (TNF-R), TNF-α converting enzyme (TACE), nuclear factor-κB (NF-KB), NF-KB inducing kinase (NIK) [27].

**Fig. (2):**
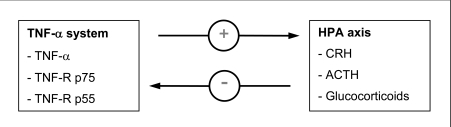
**Simplified scheme of interactions between the TNF-α system and the HPA axis.** Exogenous TNF-α administration in tumor bearing patients leads to a release of corticotropin-releasing hormone (CRH), adrenocorticotropic hormone (ACTH) and cortisol, while glucocorticoid administration in healthy subjects suppresses TNF-α production.

**Table 1.** Effects of Different Psychopharmacological Agents on Body Weight and Plasma Levels of TNF-α and its Soluble Receptors According to [7, 33, 44, 73, 91, 118]

	Body weight	TNF-α	sTNF-R p55	sTNF-R p75
Clozapine	↑↑↑	↑	↑	↑
Olanzapine	↑↑↑	↑	↑	↑
Amitriptyline	↑↑↑	↕	↑	↑
Mirtazapine	↑↑↑	↑	↑	↑
Lithium	↑↑	↑	↑	↑
Carbamazepine	↑↑	↑	↑	↑
Haloperidol	↕	↕	↕	↕
Paroxetine	↕	↕	↕	↕
Venlafaxine	↕	↕	↕	↕
Bupropion	↓	↓	n. i.	n. i.

Abreviations: increase (↑), decrease (↓), no systematical change (↕), not investigated (n. i.), tumor necrosis factor-alpha (TNF-α) , soluble TNF receptor (sTNF-R).

## References

[1] Alikhani M, Alikhani Z, Raptis M, Graves DTJ (2004). TNF-alpha *In vivo* stimulates apoptosis in fibroblasts through caspase-8 activation and modulates the expression of pro-apoptotic genes. Cell Physiol.

[2] Antonarakis SE, Blouin JL, Pulver AE, Wolyniec P, Lasseter VK, Nestadt G, Kasch L, Babb R, Kazazian HH, Dombroski B (1995). Schizophrenia susceptibility and chromosome 6p24-22. Nat. Genet.

[3] Arzt E, Kovalovsky D, Igaz LM, Costas M, Plazas P, Refojo D, Páez-Pereda M, Reul JM, Stalla G, Holsboer F (2000). Functional cross-talk among cytokines, T-cell receptor, and glucocorticoid receptor transcriptional activity and action. Ann. N. Y. Acad. Sci.

[4] Baptista T, Kin NM, Beaulieu S, de Baptista EA (2002). Obesity and related metabolic abnormalities during antipsychotic drug administration: mechanisms, management and research perspectives. Pharmacopsychiatry.

[5] Bastard JP, Maachi M, Lagathu C, Kim MJ, Caron M, Vidal H, Capeau J, Feve B (2006). Recent advances in the relationship between obesity, inflammation, and insulin resistance. Eur. Cytokine Network.

[6] Besedovsky HO, del Rey A (2000). The cytokine-HPA axis feed-back circuit. Z. Rheumatol.

[7] Brustolim D, Ribeiro-dos-Santos R, Kast RE, Altschuler EL, Soares MB (2006). A new chapter opens in anti-inflammatory treatments the antidepressant bupropion lowers production of tumor necrosis factor-alpha and interferon-gamma in mice. Int.Immunopharmacol.

[8] Carlini VP, Gaydou RC, Schiöth HB, de Barioglio SR (2007). Selective serotonin reuptake inhibitor (fluoxetine) decreases the effects of ghrelin on memory retention and food intake. Regul. Pept.

[9] Carswell EA, Old LJ, Kassel RL, Green S, Fiore N, Williamson B (1975). An endotoxin-induced serum factor that causes necrosis of tumors. Proc. Natl. Acad. Sci. USA.

[10] Choi I, Kang HS, Yang Y, Pyun KH (1994). IL-6 induces hepatic inflammation and collagen synthesis *In vivo*. Clin. Exp. Immunol.

[11] Churchill L, Taishi P, Wang M, Brandt J, Cearley C, Rehman A, Krueger JM (2006). Brain distribution of cytokine mRNA induced by systemic administration of interleukin-1beta or tumor necrosis factor alpha. Brain Res.

[12] Clark IA (2007). How TNF was recognized as a key mechanism of disease. Cytokine Growth Factor Rev.

[13] Conti B, Tabarean I, Andrei C, Bartfai T (2004). Cytokines and fever. Front. Biosci.

[14] Cummings DE, Shannon MH (2003). Roles for ghrelin in the regulation of appetite and body weight. Arch. Surg.

[15] Dantzer R (2001). Cytokine-Induced Sickness Behavior: Mechanisms and Implications. Ann. N. Y. Acad. Sci.

[16] Dauvilliers Y (2003). Neurodegenerative, autoimmune and genetic processes of human and animal narcolepsy. Rev. Neurol.

[17] Davis M (1991). Hepatotoxicity of antidepressants. Int. Clin. Psychopharmacol.

[18] Fiers W (1991). Tumor necrosis factor Characterization at the molecular, cellular and *In vivo* level. FEBS Lett.

[19] Fricchione G, Daly R, Rogers MP, Stefano GB (2001). Neuroimmunologic influences in neuropsychiatric and psychophysiologic disorders. Acta. Pharmacol. Sin.

[20] Glossop JR, Dawes PT, Nixon NB, Mattey DL (2005). Polymorphism in the tumour necrosis factor receptor II gene is associated with circulating levels of soluble tumour necrosis factor receptors in rheumatoid arthritis. Arthritis Res. Ther.

[21] Goodsell DS (2006). The molecular perspective: tumor necrosis factor. Oncologist.

[22] Guneli E, Kazikdas KC, Kolatan E (2007). Ghrelin may attenuate proinflammatory cytokine-mediated neuropathic pain. Med. Hypotheses.

[23] Haack M, Hinze-Selch D, Fenzel T, Kraus T, Kuhn M, Schuld A, Pollmächer T (1999). Plasma levels of cytokines and soluble cytokine receptors in psychiatric patients upon hospital admission: effects of confounding factors and diagnosis. J. Psychiatr. Res.

[24] Harlan J, Chen Y, Gubbins E, Mueller R, Roch JM, Walter K, Lake M, Olsen T, Metzger P, Dorwin S, Ladror U, Egan DA, Severin J, Johnson RW, Holzman TF, Voelp K, Davenport C, Beck A, Potter J, Gopalakrishnan M, Hahn A, Spear BB, Halbert DN, Sullivan JP, Abkevich V, Neff CD, Skolnick MH, Shattuck D, Katz DA (2006). Variants in Apaf-1 segregating with major depression promote apoptosome function. Mol. Psychiatry.

[25] Harris TB, Ferrucci L, Tracy RP, Corti MC, Wacholder S, Ettinger Jr WH, Heimovitz H, Cohen HJ, Wallace R (1999). Associations of elevated interleukin-6 and C-reactive protein levels with mortality in the elderly. Am. J. Med.

[26] Himmerich H (2007). Activity of the TNF-? system in patients with brain disorders and during psychopharmacological treatment. Curr. Pharmaceut. Anal.

[27] Himmerich H, Romano E, De Luca S (2008). Metabolic and neuroendocrine aspects of the TNF-? system during psychiatric disorders and psychopharmacological treatment. New Research on Neurosecretory Systems.

[28] Himmerich H, Beitinger PA, Fulda S, Wehrle R, Linseisen J, Wolfram G, Himmerich S, Gedrich K, Wetter TC, Pollmächer T (2006). Plasma levels of tumor necrosis factor alpha and soluble tumor necrosis factor receptors in patients with narcolepsy. Arch. Intern. Med.

[29] Himmerich H, Binder EB, Künzel HE, Schuld A, Lucae S, Uhr M, Pollmächer T, Holsboer F, Ising M (2006). Successful antidepressant therapy restores the disturbed interplay between TNF-alpha system and HPA axis. Biol. Psychiatry.

[30] Himmerich H, Fulda S, Künzel HE, Pfennig A, Dzaja A, Cummings DE, Pollmächer T (2005). Ghrelin plasma levels during psychopharmacological treatment. Neuropsychobiology.

[31] Himmerich H, Fulda S, Linseisen J, Seiler H, Wolfram G, Himmerich S, Gedrich K, Pollmächer T (2006). TNF-alpha, soluble TNF receptor and interleukin-6 plasma levels in the general population. Eur. Cytokine Network.

[32] Himmerich H, Kaufmann C, Schuld A, Pollmächer T (2005). Elevation of liver enzyme levels during psychopharmacological treatment is associated with weight gain. J. Psychiatr. Res.

[33] Himmerich H, Koethe D, Schuld A, Yassouridis A, Pollmächer T (2005). Plasma levels of leptin and endogenous immune modulators during treatment with carbamazepine or lithium. Psychopharmacology.

[34] Himmerich H, Schuld A, Haack M, Kaufmann C, Pollmächer T (2004). Early prediction of changes in weight during six weeks of treatment with antidepressants. J. Psychiatr. Res.

[35] Hinze-Selch D, Deuschle M, Weber B, Heuser I, Pollmächer T (2000). Effect of coadministration of clozapine and fluvoxamine versus clozapine monotherapy on blood cell counts, plasma levels of cytokines and body weight. Psychopharmacology.

[36] Hinze-Selch D, Schuld A, Kraus T, Kühn M, Uhr M, Haack M, Pollmächer T (2000). Effects of antidepressants on weight and on the plasma levels of leptin, TNF-alpha and soluble TNF receptors: A longitudinal study in patients treated with amitriptyline or paroxetine. Neuropsychopharmacology.

[37] Hohjoh H, Nakayama T, Ohashi J, Miyagawa T, Tanaka H, Akaza T, Honda Y, Tokunaga K (1999). Significant association of a single nucleotide polymorphism in the tumor necrosis factor-alpha (TNF-alpha) gene promoter with human narcolepsy. Tissue Antigens.

[38] Hornig M, Lipkin WI (2001). Infectious and immune factors in the pathogenesis of neurodevelopmental disorders epidemiology, hypotheses, and animal models. Ment. Retard. Dev. Disabil. Res. Rev.

[39] Hoshi T, Kitagawa K, Yamagami H, Furukado S, Hougaku H, Hori M (2005). Relations of serum high-sensitivity C-reactive protein and interleukin-6 levels with silent brain infarction. Stroke.

[40] Ikejima K, Takei Y, Honda H, Hirose M, Yoshikawa M, Zhang YJ, Lang T, Fukuda T, Yamashina S, Kitamura T, Sato N (2002). Leptin receptor-mediated signaling regulates hepatic fibrogenesis and remodeling of extracellular matrix in the rat. Gastroenterology.

[41] Jenny NS, Tracy RP, Ogg MS, Luong A, Kuller LH, Arnold AM, Sharrett AR, Humphries SE (2002). In the elderly, interleukin-6 plasma levels and the -174G > C polymorphism are associated with the development of cardiovascular disease. Arterioscler. Thromb. Vasc. Biol.

[42] Kapcala LP, Chautard T, Eskay RL (1995). The protective role of the hypothalamic-pituitary-adrenal axis against lethality produced by immune, infectious, and inflammatory stress. Ann. N. Y. Acad. Sci.

[43] Karni A, Abraham M, Monsonego A, Cai G, Freeman GJ, Hafler D, Khoury SJ, Weiner HL (2006). Innate immunity in multiple sclerosis: myeloid dendritic cells in secondary progressive multiple sclerosis are activated and drive a proinflammatory immune response. J. Immunol.

[44] Kraus T, Haack M, Schuld A, Hinze-Selch D, Koethe D, Pollmächer T (2002). Body weight, the tumor necrosis factor system, and leptin production during treatment with mirtazapine or venlafaxine. Pharmacopsychiaty.

[45] Kraus T, Haack M, Schuld A, Hinze-Selch D, Kühn M, Uhr M, Pollmächer T (1999). Body weight and leptin plasma levels during treatment with antipsychotic drugs. Am. J. Psychiatry.

[46] Ledeen RW, Chakraborty G (1998). Cytokines, signal transduction, and inflammatory demyelination: review and hypothesis. Neurochem. Res.

[47] van der Lely AJ, Tschöp M, Heiman ML, Ghigo E (2004). Biological, physiological, pathophysiological, and pharmacological aspects of ghrelin. Endocr. Rev.

[48] Lin L, Hungs M, Mignot E (2001). Narcolepsy and the HLA region. J. Neuroimmunol.

[49] Lindholm E, Ekholm B, Balciuniene J, Johansson G, Castensson A, Koisti M, Nylander PO, Pettersson U, Adolfsson R, Jazin E (1999). Linkage analysis of a large Swedish kindred provides further support for a susceptibility locus for schizophrenia on chromosome 6p23. Am. J. Med. Genet.

[50] Linthorst AC, Reul JM (1999). Inflammation and brain function under basal conditions and during long-term elevation of brain corticotropin-releasing hormone levels. Adv. Exp. Med. Biol.

[51] Maes M, Scharpé S, Meltzer HY, Bosmans E, Suy E, Calabrese J, Cosyns P (1993). Relationships between interleukin-6 activity, acute phase proteins, and function of the hypothalamic-pituitary-adrenal axis in severe depression. Psychiatry Res.

[52] Maes M, Song C, Lin AH, Bonaccorso S, Kenis G, De Jongh R, Bosmans E, Scharpe S (1999). Negative immunoregulatory effects of antidepressants: inhibition of interferon-gamma and stimulation of interleukin-10 secretion. Neuropsychopharmacology.

[53] Maes M, Song C, Lin AH, Pioli R, Kenis G, Kubera M, Bosmans E (1999). *In vitro* immunoregulatory effects of lithium in healthy volunteers. Psychopharmacology.

[54] Merrill JE (1992). Proinflammatory and anti-inflammatory cytokines in multiple sclerosis and central nervous system acquired immunodeficiency syndrome. J. Immunother.

[55] Michie HR, Spriggs DR, Manogue KR, Sherman ML, Revhaug A, O'Dwyer ST, Arthur K, Dinarello CA, Cerami A, Wolff SM, Wilmom DW (1988). Tumor necrosis factor and endotoxin induce similar metabolic responses in human beings. Surgery.

[56] Mignot E, Tafti M, Dement WC, Grumet FC (1995). Narcolepsy and immunity. Adv. Neuroimmunol.

[57] Möller E, Böhme J, Valugerdi MA, Ridderstad A, Olerup O (1990). Speculations on mechanisms of HLA associations with autoimmune diseases and the specificity of "autoreactive" T lymphocytes. Immunol. Rev.

[58] Murashita M, Kusumi I, Hosoda H, Kangawa K, Koyama T (2007). Acute administration of clozapine concurrently increases blood glucose and circulating plasma ghrelin levels in rats. Psychoneuroendocrinology.

[59] Murashita M, Kusumi I, Inoue T, Takahashi Y, Hosoda H, Kangawa K, Koyama T (2005). Olanzapine increases plasma ghrelin level in patients with schizophrenia. Psychoneuroendocrinology.

[60] Nakazato M, Murakami N, Date Y, Kojima M, Matsuo H, Kangawa K, Matsukura S (2001). A role for ghrelin in the central regulation of feeding. Nature.

[61] Newton JR (2007). Linked gene ontology categories are novel and differ from associated gene ontology categories for the bipolar disorders. Psychiatr. Genet.

[62] Nishino S, Kanbayashi T (2005). Symptomatic narcolepsy, cataplexy and hypersomnia, and their implications in the hypothalamic hypocretin/orexin system. Sleep Med. Rev.

[63] O'Brien SM, Scott LV, Dinan TG (2004). Cytokines abnormalities in major depression and implications for pharmacological treatment. Hum. Psychopharmacol.

[64] Panagiotakos DB, Pitsavos C, Yannakoulia M, Chrysohoou C, Stefanadis C (2005). The implication of obesity and central fat on markers of chronic inflammation: The ATTICA study. Atherosclerosis.

[65] Park HS, Park JY, Yu R (2005). Relationship of obesity and visceral adiposity with serum concentrations of CRP, TNF-alpha and IL-6. Diabetes Res. Clin. Pract.

[66] Pedersen M, Bruunsgaard H, Weis N, Hendel HW, Andreassen BU, Eldrup E, Dela F, Pedersen BK (2003). Circulating levels of TNF-alpha and IL-6-relation to truncal fat mass and muscle mass in healthy elderly individuals and in patients with type-2 diabetes. Mech. Ageing Dev.

[67] Perskidskii IuV, Barshtein IuA (1992). Biological manifestations of the tumor necrosis factor effect and its role in the pathogenesis of various diseases. Arkh. Patol.

[68] Pessayre D, Berson A, Fromenty B, Mansouri A (2001). Mitochondria in steatohepatitis. Semin. Liver Dis.

[69] Petrovsky N, McNair P, Harrison LC (1998). Diurnal rhythms of pro-inflammatory cytokines regulation by plasma cortisol and therapeutic implications. Cytokine.

[70] Pickering M, Cumiskey D, O'Connor JJ (2005). Actions of TNF-? on glutamatergic synaptic transmission in the central nervous system. Exp. Physiol.

[71] Plata-Salamán CR (1999). Brain mechanisms in cytokine-induced anorexia. Psychoneuroendocrinology.

[72] Plumpe J, Malek NK, Bock CT, Rakemann T, Manns MP, Trautwein C (2000). NF-kappaB determines between apoptosis and proliferation in hepatocytes during liver regeneration. Am. J. Physiol. Gastrointest. Liver Physiol.

[73] Pollmächer T, Haack M, Schuld A, Kraus T, Hinze-Selch D (2000). Effects of antipsychotic drugs on cytokine networks. J. Psychiatr. Res.

[74] Pollmächer T, Hinze-Selch D, Fenzel T, Kraus T, Schuld A, Mullington J (1997). Plasma levels of cytokines and soluble cytokine receptors during treatment with haloperidol. Am. J. Psychiatry.

[75] Pollmächer T, Hinze-Selch D, Mullington J (1996). Effects of clozapine on plasma cytokine and soluble cytokine receptor levels. J. Clin. Psychopharmacol.

[76] Pucak ML, Kaplin AI (2005). Unkind cytokines: current evidence for the potential role of cytokines in immune-mediated depression. Int. Rev. Psychiatry.

[77] Reddy P, Slack JL, Davis R, Cerretti DP, Kozlosky CJ, Blanton RA, Shows D, Peschon JJ, Black RAJ (2000). Functional analysis of the domain structure of tumor necrosis factor-alpha converting enzyme. Biol. Chem.

[78] Reichenberg A, Kraus T, Haack M, Schuld A, Pollmächer T, Yirmiya R (2002). Endotoxin-induced changes in food consumption in healthy volunteers are associated with TNF-alpha and IL-6 secretion. Psychoneuroendocrinology.

[79] Reichenberg A, Yirmiya R, Schuld A, Kraus T, Haack M, Morag A, Pollmächer T (2001). Cytokine-associated emotional and cognitive disturbances in humans. Arch Gen. Psychiatry.

[80] Reul JM, Labeur MS, Wiegers GJ, Linthorst AC (1998). Altered neuroimmunoendocrine communication during a condition of chronically increased brain corticotropin-releasing hormone drive. Ann. N. Y. Acad. Sci.

[81] Ridker PM, Rifai N, Stampfer MJ, Hennekens CH (2000). Plasma concentration of interleukin-6 and the risk of future myocardial infarction among apparently healthy men. Circulation.

[82] Roerig JL, Steffen KJ, Mitchell JE, Crosby RD, Gosnell BA (2008). A comparison of the effects of olanzapine and risperidone versus placebo on ghrelin plasma levels. J. Clin. Psychopharmacol.

[83] Schmid DA, Wichniak A, Uhr M, Ising M, Brunner H, Held K, Weikel JC, Sonntag A, Steiger A (2006). Changes of sleep architecture, spectral composition of sleep EEG, the nocturnal secretion of cortisol, ACTH, GH, prolactin, melatonin, ghrelin, and leptin, and the DEX-CRH test in depressed patients during treatment with mirtazapine. Neuropsychopharmacology.

[84] Schöbitz B, Reul JM, Holsboer F (1994). The role of the hypothalamic-pituitary-adrenocortical system during inflammatory conditions. Crit. Rev. Neurobiol.

[85] Schuld A, Kraus T, Haack M, Hinze-Selch D, Kühn M, Pollmächer T (2000). Plasma levels of cytokines and soluble cytokine receptors during treatment with olanzapine. Schizophr. Res.

[86] Schuld A, Schmid DA, Haack M, Holsboer F, Friess E, Pollmächer T (2003). Hypothalamo-pituitary-adrenal function in patients with depressive disorders is correlated with baseline cytokine levels, but not with cytokine responses to hydrocortisone. J. Psychiatr. Res.

[87] Schwab SG, Albus M, Hallmayer J, Honig S, Borrmann M, Lichtermann D, Ebstein RP, Ackenheil M, Lerer B, Risch N (1995). Evaluation of a susceptibility gene for schizophrenia on chromosome 6p by multipoint affected sib-pair linkage analysis. Nat. Genet.

[88] Selim K, Kaplowitz N (1999). Hepatotoxicity of psychotropic drugs. Hepatology.

[89] Silverman MN, Pearce BD, Biron CA, Miller AH (2005). Immune modulation of the hypothalamic-pituitary-adrenal (HPA) axis during viral infection. Viral Immunol.

[90] Sorge S (2003). Der Einfluss des atypischen Neuroleptikums Clozapin auf das Verhalten und das Immunsystem der Ratte. Dissertationsschrift München. Technische Universitt Mnchen.

[91] Spies T, Morton CC, Nedospasov SA, Fiers W, Pious D, Strominger JL (1986). Genes for the tumor necrosis factors alpha and beta are linked to human major histocompatibility complex. Proc. Natl. Acad. Sci. USA.

[92] Takeuchi H, Jin S, Wang J, Zhang G, Kawanokuchi J, Kuno R, Sonobe Y, Mizuno T, Suzumura A (2006). Tumor necrosis factor-alpha induces neurotoxicity *via* glutamate release from hemichannels of activated microglia in an autocrine manner. J. Biol. Chem.

[93] Tanabe A, Nomura S (2007). Pathophysiology of depression. Nippon Rinsho.

[94] Thannickal TC, Moore RY, Nienhuis R, Ramanathan L, Gulyani S, Aldrich M, Cornford M, Siegel JM (2000). Reduced number of hypocretin neurons in human narcolepsy. Neuron.

[95] Theisen FM, Gebhardt S, Brömel T, Otto B, Heldwein W, Heinzel-Gutenbrunner M, Krieg JC, Remschmidt H, Tschöp M, Hebebrand J (2005). A prospective study of serum ghrelin levels in patients treated with clozapine. J. Neural. Transm.

[96] Tilders FJ, DeRijk RH, Van Dam AM, Vincent VA, Schotanus K, Persoons JH (1994). Activation of the hypothalamus-pituitary-adrenal axis by bacterial endotoxins: routes and intermediate signals. Psychoneuroendocrinology.

[97] Tilg H (2001). Cytokines and liver diseases. Can. J. Gastroenterol.

[98] Tschöp M, Smiley DL, Heiman ML (2000). Ghrelin induces adiposity in rodents. Nature.

[99] Tschöp M, Weyer C, Tataranni PA, Devanarayan V, Ravussin E, Heiman ML (2001). Circulating ghrelin levels are dcreased in human obesity. Diabetes.

[100] Tyring S, Gottlieb A, Papp K, Gordon K, Leonardi C, Wang A, Lalla D, Woolley M, Jahreis A, Zitnik R, Cella D, Krishnan R (2006). Etanercept and clinical outcomes, fatigue, and depression in psoriasis: double-blind placebo-controlled randomised phase III trial. Lancet.

[101] Wajant H, Pfizenmaier K, Scheurich P (2003). Tumor necrosis factor signaling. P. Cell Death Differ.

[102] Waje-Andreassen U, Krakenes J, Ulvestad E, Thomassen L, Myhr KM, Aarseth J, Vedeler CA (2005). IL-6: an early marker for outcome in acute ischemic stroke. Acta Neurol. Scand.

[103] Wang S, Sun CE, Walczak CA, Ziegle JS, Kipps BR, Goldin LR, Diehl SR (1995). Evidence for a susceptibility locus for schizophrenia on chromosome 6pter-p22. Nat. Genet.

[104] Waseem T, Duxbury M, Ito H, Ashley SW, Robinson MK (2008). Exogenous ghrelin modulates release of pro-inflammatory and anti-inflammatory cytokines in LPS-stimulated macrophages through distinct signaling pathways. Surgery.

[105] Weinberger DR, Lipska BK (1995). Cortical maldevelopment, antipsychotic drugs, and schizophrenia: a search for common ground. Schizophr. Res.

[106] Wichers M, Maes M (2002). The psychoneuroimmuno-patho-physiology of cytokine-induced depression in humans. Int. J. Neuropsychopharmacol.

[107] Wilson AG, Symons JA, McDowell TL, McDevitt HO, Duff GW (1997). Effects of a polymorphism in the human tumor necrosis factor alpha promoter on transcriptional activation. Proc. Natl. Acad. Sci. USA.

[108] Wren AM, Seal LJ, Cohen MA, Brynes AE, Frost GS, Murphy KG, Dhillo WS, Ghatei MA, Bloom SR (2001). Grelin enhances appetite and increases food intake in humans. J. Clin. Endocrinol. Metab.

[109] Wren AM, Small CJ, Abbott CR, Dhillo WS, Seal LJ, Cohen MJ, Batterham RL, Taheri S, Stanley SA, Ghatei MA, Bloom SR (2001). Ghrelin causes hyperphagia and obesity in rats. Diabetes.

[110] Wu R, Dong W, Cui X, Zhou M, Simms HH, Ravikumar TS, Wang P (2007). Ghrelin down-regulates proinflammatory cytokines in sepsis through activation of the vagus nerve. Ann. Surg.

[111] Wu R, Dong W, Zhou M, Zhang F, Marini CP, Ravikumar TS, Wang P (2007). Ghrelin attenuates sepsis-induced acute lung injury and mortality in rats. Am. J. Respir. Crit. Care Med.

[112] Yirmiya R (1997). Behavioral and psychological effects of immune activation: implications for 'depression due to a general medical condition. Curr. Opin. Psychiatry.

[113] Zai G, Müller DJ, Volavka J, Czobor P, Lieberman JA, Meltzer HY, Kennedy JL (2006). Family and case-control association study of the tumor necrosis factor-alpha (TNF-alpha) gene with schizophrenia and response to antipsychotic medication. Psychopharmacology.

[114] Zeyda M, Farmer D, Todoric J, Aszmann O, Speiser M, Györi G, Zlabinger GJ, Stulnig TM (2007). Human adipose tissue macrophages are of an anti-inflammatory phenotype but capable of excessive pro-inflammatory mediator production. Int. J. Obes.

[115] Zhang H, Yin J, Li D, Zhou X, Li X (2007). Tryptophan enhances ghrelin expression and secretion associated with increased food intake and weight gain in weanling pigs. Domest. Anim. Endocrinol.

[116] Zhang M, Yuan F, Chen H, Qiu X, Fang W (2007). Effect of exogenous ghrelin on cell differentiation antigen 40 expression in endothelial cells. Acta Biochim. Biophys. Sin.

[117] Zhu CB, Blakely RD, Hewlett WA (2006). The proinflammatory cytokines interleukin-1beta and tumor necrosis factor-alpha activate serotonin transporters. Neuropsychopharmacology.

[118] Zimmermann U, Kraus T, Himmerich H, Schuld A, Pollmächer T (2003). Epidemiology, implications and mechanisms underlying drug-induced weight gain in psychiatric patients. J. Psychiatr. Res.

